# Heterogeneity of predictive biomarker expression in gastric and esophago-gastric junction carcinoma with peritoneal dissemination

**DOI:** 10.1007/s10120-025-01609-7

**Published:** 2025-04-09

**Authors:** Valentina Angerilli, Matilde Callegarin, Ilaria Govoni, Giuseppe De Lisi, Michele Paudice, Paola Fugazzola, Alessandro Vanoli, Paola Parente, Francesca Bergamo, Claudio Luchini, Angelo Paolo Dei Tos, Federica Grillo, Sara Lonardi, Luca Mastracci, Gaya Spolverato, Matteo Fassan

**Affiliations:** 1 Department of Medicine (DIMED), Surgical Pathology & Cytopathology Unit, University of Padua, via Gabelli 61, 35121 Padua, Italy; 2 ULSS2 Marca Trevigiana, Treviso, Italy; 3 Department of Pathology, Radboud Institute for Molecular Life Sciences, Radboud University Medical Centre, Nijmegen, The Netherlands; 4 Department of Surgical, Oncological and Gastroenterological Sciences (DiSCOG), University of Padua, Padua, Italy; 5 IRCCS San Matteo Hospital, Pavia, Italy; 6 Anatomic Pathology Unit, Department of Molecular Medicine, University of Pavia, Pavia, Italy; 7 Anatomic Pathology, Department of Surgical Sciences and Integrated Diagnostics (DISC), University of Genova, Genoa, Italy; 8 IRCCS Ospedale Policlinico San Martino, Genoa, Italy; 9 General Surgery Unit, Fondazione IRCCS Policlinico San Matteo, Pavia, Italy; 10 Unit of Pathology, Fondazione IRCCS Ospedale Casa Sollievo della Sofferenza, San Giovanni Rotondo, Italy; 11 Veneto Institute of Oncology IOV—IRCCS, Padua, Italy; 12 Department of Pathology and Diagnostics, University and Hospital Trust of Verona, Verona, Italy

**Keywords:** Gastroesophageal cancer, Biomarkers, Heterogeneity, Targeted therapy

## Abstract

**Background:**

Temporal and spatial molecular heterogeneity contributes to resistance to targeted and immune therapies in gastric and esophagogastric junction carcinoma (G/EGJ). This study evaluates differences in biomarker expression between primary G/EGJ and paired peritoneal metastases (PM).

**Methods:**

We analyzed 74 cases of primary G/EGJ and paired PM using immunohistochemistry for HER2, PD-L1, Claudin18 (CLDN18), DNA mismatch repair (MMR) proteins, p53, E-cadherin, and in situ hybridization for EBER. Biomarker concordance between primary and metastatic tumors was assessed.

**Results:**

Primary G/EGJ were predominantly poorly cohesive (45.9%) or mixed-type (37.8%). Regarding predictive biomarkers, low rates of HER2 overexpression (5.4%), MMR deficiency (4.1%), and EBER positivity (1.4%) were observed, while PD-L1 CPS ≥ 1 occurred in 79.7% of cases and CLDN18 positivity was observed in 31.1% of cases. Concordance was perfect for MMR and EBER, while PD-L1 showed the highest discordance (32.4%). HER2 had a low discordance rate (2.7%). CLDN18 exhibited good concordance (86.5%) and showed consistent positivity in PD-L1- and HER2-negative primary tumors (28.6%).

**Conclusion:**

G/EGJ with PM show distinct molecular features and spatial heterogeneity, with MMR, EBER, and HER2 demonstrating strong concordance, while PD-L1 showed greater variability. As for novel biomarkers, CLDN18.2 shows substantial concordance between primary G/EGJ and PM and could be a promising target in HER2/PD-L1-negative G/EGJ with PM.

**Supplementary Information:**

The online version contains supplementary material available at 10.1007/s10120-025-01609-7.

## Introduction

Gastric and esophagogastric junction carcinoma (G/EGJ), encompassing gastric and esophageal junction cancers, represent a significant health burden and are among the leading causes of cancer-related deaths worldwide. G/EGJ are often diagnosed at an advanced stage with peritoneum being a frequent site of metastasis. PM can be found at the initial G/EGJ diagnosis or during intended radical surgery (synchronous PM). Following radical surgery, PM occurs as peritoneal recurrence (metachronous PM), accounting for up to 50% of all recurrences [1, 2]. Risk factors for peritoneal dissemination include advanced primary T or N stage, female sex, young age, non-cardia gastric cancer, diffuse type histology and *linitis plastica* [3, 4]. Peritoneal implants are now being considered as locally disseminated disease and patients with limited peritoneal involvement can be treated by combining systemic chemotherapy, cytoreductive surgery, and hyperthermic intraperitoneal chemotherapy [5]. However, the prognosis of patients with peritoneal carcinosis remains poor.

In the era of precision oncology, the global efforts undertaken to decipher the molecular heterogeneity of G/EGJ have led to the development of several targeted and immune therapies and the identification of predictive and prognostic biomarkers [6]. Previous reports have indicated that G/EGJ with PM exhibit low rates of microsatellite instability (MSI) and HER2 overexpression [7]. Furthermore, the clinical efficacy of immune checkpoint inhibitors (ICIs) against G/EGJ with PM is still unclear. Earlier studies have suggested that the response to PD-1 inhibitors tended to be less prominent in G/EGJ with PM [8, 9]. The introduction of novel therapies into clinical practice, including claudin inhibitors and trastuzumab deruxtecan, might broaden the range of therapeutic options for G/EGJ with PM [10, 11]. Despite the growing array of therapeutic targets, systemic treatments frequently fall short in addressing PM due to inadequate drug delivery. For this reason, future perspectives include intraperitoneal delivery of targeted and immune therapy [12].

It is well established that intra-tumoral spatial molecular heterogeneity may impact responsiveness and fuel resistance to personalized therapies. Significant genomic and transcriptomic differences can be found between primary and metastatic G/EGJ, with distinct molecular signatures pinpointed for peritoneal dissemination [13, 14]. These differences may pose challenges to the effectiveness of both systemic and intraperitoneal therapies. This study aims to explore the expression patterns of both established and emerging predictive biomarkers in G/EGJ with PM and to investigate the consistency of biomarker expression between the primary tumor and PM.

## Materials and methods

### Study cohort

A cohort of 74 patients diagnosed between 2012 and 2022 with G/EGJ with synchronous or metachronous PM were included in this multicentric study. Specimens of primary G/EGJ and matched PM samples were collected from the surgical pathology units of Padua University Hospital (Padua, Italy), Ospedale Policlinico San Martino IRCCS (Genoa, Italy), Fondazione IRCCS Casa Sollievo della Sofferenza (San Giovanni Rotondo, Italy) and Fondazione IRCCS Policlinico San Matteo (Pavia, Italy).

Clinical and pathologic data on sex, age, primary tumor site and neoadjuvant treatment were collected from the pathology reports and clinical records. Primary tumor samples were biopsy specimens in four cases and surgical resection specimens in the remaining. PM were always peritoneal nodules.

In all patients, Hematoxylin and Eosin (HE) slides obtained from the formalin-fixed paraffin-embedded (FFPE) tissue samples were jointly reviewed by three expert gastrointestinal pathologists (FG, PP, MF) to establish pTNM stage (according to the 8th edition American Joint Committee on Cancer [AJCC] staging system criteria [15]), histotype and grade (according to the 5th edition of WHO digestive tumors classification [16]).

All information regarding human material was managed using anonymous numerical codes, and all samples were handled in compliance with the Declaration of Helsinki.

### Immunohistochemical (IHC) analysis

Immunohistochemical (IHC) stains were performed on primary G/EGJ and paired PM using the Bond Polymer Refine Detection Kit (Leica Biosystems, Newcastle upon Tyne, UK) on BOND-MAX automated IHC stainer (Leica Biosystems, Newcastle upon Tyne, UK). Four μm thick FFPE section was incubated with the following primary antibodies according to the laboratories routine practice: p53 (clone DO-7; Dako), E-cadherin (NCH-38; Dako), PM2 (EP51; Dako), MSH6 (EP49; Dako), HER2 (4B5; Ventana), PD-L1 (22C3; Dako), CLDN18, (clone 43-14A; Roche Ventana). One IHC slide per marker was assessed from a single FFPE tissue block.

E-cadherin expression was considered altered in the presence of complete loss or markedly reduced membranous staining (< 30%), regardless of nuclear/cytoplasmic staining.

p53 was considered aberrant in the presence of complete loss or diffuse and strong nuclear immunostaining in neoplastic cells.

Mismatch Repair defective status was assessed by testing PM2 and MSH6, and samples were defined as deficient (MMRd) when one or both proteins resulted in negative. In case of protein loss, the dominant component of the heterodimer (i.e., MLH1 for PM2 and MSH2 for MSH6; Dako) was tested.

For the evaluation of HER2, the four-tier Hoffmann scoring criteria were used. In surgical specimens, when complete or basolateral membranous reactivity was observed in ≥ 10% of tumor cells, scores of 1 + , 2 + and 3 + are determined according to the intensity of membranous reactivity (faint, moderate or intense). In biopsy specimens, when membranous reactivity was observed in at least one cancer cell cluster (≥ 5 cells), scores of 1 + , 2 + and 3 + were determined according to its intensity (faint, moderate or intense). Equivocal cases (i.e., HER2 2 +) were analyzed by HER2 fluorescent in situ hybridization (FISH) to test for gene amplification. HER2 was classified as either overexpressed/not overexpressed or zero/low/high. HER2 0, HER2 1 + and not-amplified 2 + cases were considered as HER2-not overexpressed, HER2 1 + and not-amplified 2 + cases were classified as HER2-low and HER2 amplified 2 + and 3 + cases were classified as HER2-overexpressed or HER2-high.

PD-L1 expression was evaluated by using the Combined Positive Score (CPS). CPS 1 and 10 were used as cut-offs values.

CLDN18 immunoreactivity was evaluated with a quantitative (percentage of stained tumor cells method. Tumors with a 2 + /3 + score of CLDN18 intensity in ≥ 75% of tumor cells, which is the IHC cutoff being used for eligibility in ongoing zolbetuximab clinical trials, were considered positive for CLDN18 [17].

The presence and pattern of intratumoral heterogeneity were assessed in HER2-overexpressed and CLDN18-positive cases, as weak and patchy staining makes it difficult to assess staining patterns. The absence of intratumoral heterogeneity (i.e., homogenous pattern of expression) was defined as CLDN18 being expressed in more than 90% of the tumor cells with a moderate-to-strong membranous staining and as HER2 intense complete/basolater/lateral staining in more than 90% of the tumor cells. The heterogeneous pattern of expression for HER2 and CLDN18 was further classified into three subtypes: superficial, invasive-front, and random. The superficial pattern was defined by expression predominantly located in the mucosa. The invasive-front pattern showed prominent expression in the deep invasive areas of the tumor. The random pattern was characterized by patchy expression with varying intensities distributed evenly. The type of pattern of intratumoral heterogeneity was assessed only for surgical specimens.

### EBER in situ hybridization (ISH)

The Bond ready-to-use ISH Epstein-Barr virus-encoded small RNA (EBER) Probe was used in a Leica Bond-Max automation system according to the manufacturer’s instructions (Leica Biosystems) to detect Epstein–Barr virus (EBV) infection.

### HER2 fluorescent in situ hybridization (FISH)

FISH was performed according to the manufacturer’s protocol with the PathVysion HER2 DNA Probe kit and a HER2/CEP17 probe mix (Vysis; Downers Grove, Illinois, USA) and was analyzed by using a fluorescence microscope (BX61; Olympus, Hicksville, New York, USA); image capture was performed with CytoVision V.3.93 software (Applied Imaging, Pittsburgh, Pennsylvania, USA).

### Statistical analysis

The discordance in morphology, molecular features and expression of predictive biomarkers between primary tumors and paired PM was calculated as the ratio of discordant cases to total cases. Confidence intervals for discordance rates were calculated. Cohen’s κ coefficient was used to evaluate the strength of agreement between primary tumors and paired PM for each parameter. Chi-square and Fisher’s exact tests were used, where appropriate. Two-tailed P-values of 0.05 or less were considered to have a statistical significance. Statistical analysis was performed using Python 3.10.

## Results

### Clinico-pathological features

A total of 74 patients were included in this study, 34 (45.9%) females and 40 (54.1%) males (male to female ratio = 1.2:1), with a median age at cancer diagnosis of 66.5 years (range, 29–89). The majority of cases were of antro-angular localization (58.1%). The most frequent histotype was the poorly cohesive (45.9%) followed by the mixed (37.8%) histotype. Poorly cohesive G/EGJ were classified as PC-NOS (*n* = 19), PC-SRC (*n* = 9) and PC-NOS/SRC (*n* = 6) [18]. Most cases were pT4a (55.7%) for tumor extent and pN3b (29.0%) for lymph node involvement. Fourteen patients (18.9%) received neoadjuvant therapy. The clinico-pathological features of the study cohort are reported in Supplementary Table 1.

### Molecular features and expression of predictive biomarkers of primary gastric and esophago-gastric junction carcinomas with peritoneal dissemination

Overall, 50.0% of primary G/EGJ had aberrant p53 expression, showing either a clonal (*n* = 31) or null (*n* = 6) phenotype. E-Cadherin was lost in 18.9% of cases, encompassing 13 poorly cohesive carcinomas and the poorly cohesive component of one mixed carcinoma.

As for established predictive biomarkers, only 4.1% of cases were MMRd and 5.4% were HER2-overexpressed (score 3 +). PD-L1 was expressed (CPS ≥ 1) in 79.7% of primary G/EGJ. However, only 14.9% were CPS ≥ 10. As for emerging biomarkers,14.9% of cases were HER2-low, 31.1% were CLDN18 positive and only 1.4% were EBV-associated. Of note, no difference in biomarker staining pattern between the two components was observed in G/EGJ of mixed histotypes.

We used two previously published algorithms [19] for approximating The Cancer Genome Atlas (TCGA) subtypes and Asian Cancer Research Group (ACRG) subtypes using IHC/ISH and morphology. According to TCGA classification, primary tumors were classified as Genomically Stable (GS) in 33 cases (44.6%), Chromosomally Instable (CIN) in 37 cases (50.0%), Microsatellite Instable (MSI) in 3 cases (4.1%) and EBV-associated in one case (1.4%). When using the ACRG classification, primary tumors were classified as Microsatellite Stable/Epithelial Mesenchymal Transition (MSS/EMT) in 33 cases (44.6%), Microsatellite Stable/*TP53* active (MSS/TP53 +) in 21 cases (28.4%), Microsatellite Stable/*TP53* inactive (MSS/TP53-) in 17 cases (23.0%), and MSI in 3 cases (4.1%).

**Fig. 1:**
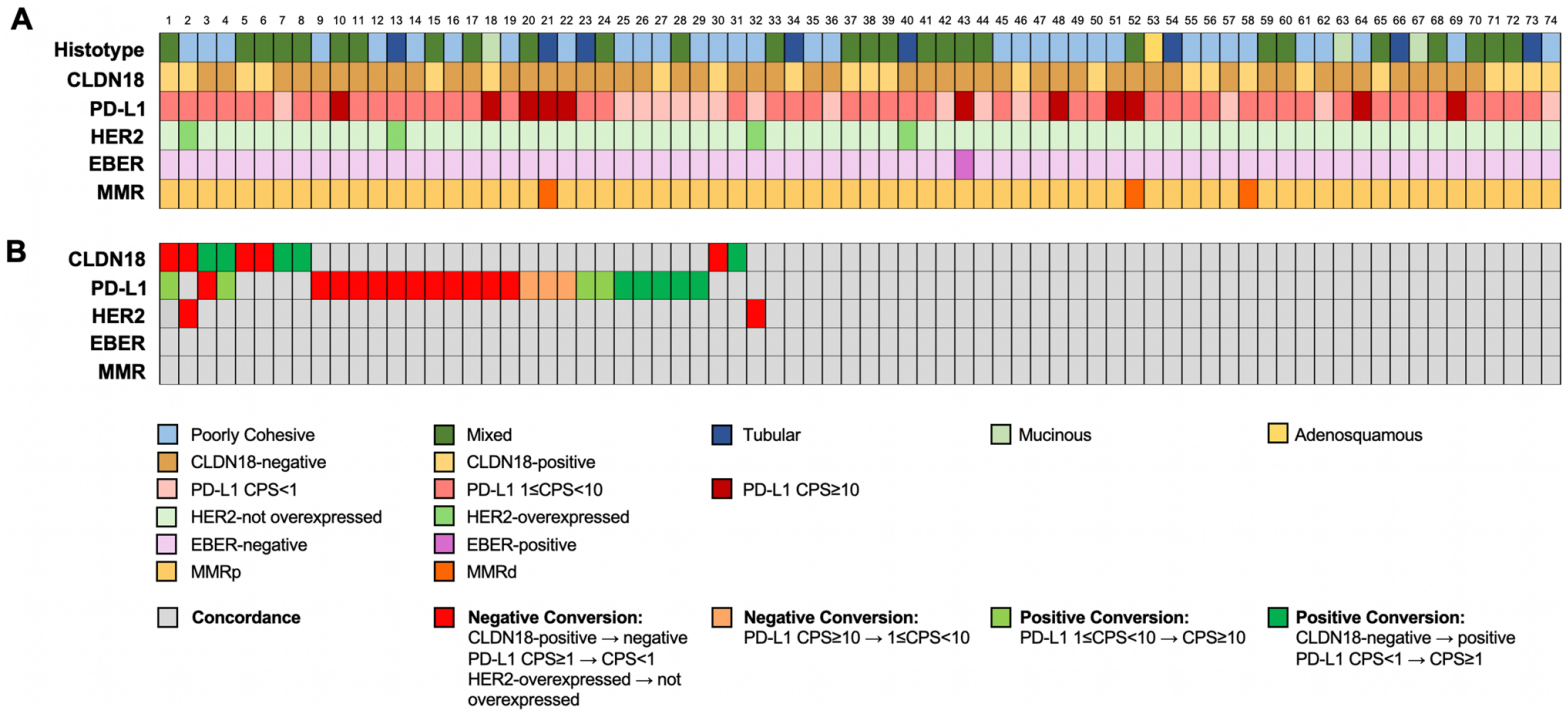
**A** Histotype and status of predictive biomarkers in gastric and esophago-gastric junction carcinomas with paired peritoneal metastases and **B** combinations of concordant and discordant biomarkers in each patient (*n* = 74). Cases with both synchronous and metachronous peritoneal metastases (PM) were considered only once; when the expression differed between synchronous and metachronous (PM), the discordant case was considered. *CPS* Combined Positive Score, *CLDN18* Claudin18, *EBER* Epstein–Barr virus–encoded small RNA, *PD-L1* programmed cell death ligand 1, *MMRd* mismatch repair deficient, *MMRp* mismatch repair proficient

The molecular features and expression of predictive biomarkers of primary gastric and esophago-gastric junction carcinomas with PM are reported in Table 1 and shown in Fig. 1A.

**Table 1:** Molecular features and expression of predictive biomarkers in primary gastric and esophago-gastric junction carcinomas (*n* = 74) with peritoneal dissemination

p53	Aberrant	37/74 (50.0%)
	- Clonal - Null	31/74 (41.9%) 6/74 (8.1%)
	Wild-type	37/74 (50.0%)
E-Cadherin	Lost	14/74 (18.9%)
	Retained	60/74 (81.1%)
MMR	MMRd	3/74 (4.1%)
	MMRp	71/74 (95.9%)
HER2	Overexpressed	4/74 (5.4%)
	Not overexpressed	70/74 (94.6%)
HER2	High	4/74 (5.4%)
	Low	11/74 (14.9%)
	0	59/74 (79.7%)
EBER	Positive	1/74 (1.4%)
	Negative	73/74 (98.6%)
PD-L1 (CPS)	CPS < 1	15/74 (20.3%)
	1 ≤ CPS < 10	48/74 (64.9%)
	CPS ≥ 10	11/74 (14.9%)
CLDN18	Positive	23/74 (31.1%)
	Negative	51/74 (68.9%)
TCGA class	EBV	1/74 (1.4%)
	MSI	3/74 (4.1%)
	GS	33/74 (44.6%)
	CIN	37/74 (50.0%)
ACRG class	MSI	3/74 (4.1%)
	MSS/EMT	33/74 (44.6%)
	MSS/TP53 +	21/74 (28.4%)
	MSS/TP53-	17/74 (23.0%)

*MMR* Mismatch Repair, *MMRp* MMR proficient, *MMRp* MMR deficient, *CPS* Combined Positive Score, *CLDN18* Claudin 18, *EBER* Epstein–Barr virus–encoded small RNA, *PD-L1* programmed cell death ligand 1, *TCGA* The Cancer Genome Atlas, *MSI* Microsatellite Instability, *GS* Genomically Stable, *CIN* Chromosomal Instability, *ACRG* Asian Cancer Research Group, *MSS* Microsatellite Stability, *EMT* Epithelial Mesenchymal Transition

### Intratumoral heterogeneity of CLDN18 and HER2 in primary G/EGJ

Among the 23 CLDN18-positive primary G/EGJ cases, the majority (16/23; 69.6%) exhibited a homogeneous expression pattern. The remaining 7 cases (30.5%) demonstrated heterogeneous expression. Of these, five cases displayed a random pattern of heterogeneity, one case showed an invasive-front pattern, and in one case of adenosquamous carcinoma, CLDN18 expression was restricted to the adenocarcinoma component.

Among the four HER2-overexpressed primary G/EGJ cases, one exhibited a homogeneous expression pattern, while the remaining three displayed heterogeneous patterns. Of these, two cases had a random heterogeneity pattern, and one case showed superficial positivity.

### Comparison of morphology, molecular features and expression of predictive biomarkers between primary gastric and esophago-gastric junction carcinomas and paired PM

Overall, synchronous PM were available for analysis in 60 cases, while metachronous PM were available for analysis in 10 cases. Additionally, in 4 cases, both synchronous and metachronous PM were present.

As for histotype, similar discordance rates were found for synchronous PM (21.9%) and for metachronous PM (28.6%). Discordance was observed only in primary tumors with mixed or mucinous histotype. In 15 out of 28 primary mixed GCs only the PCC (11/15) or the tubular component (4/15) metastasized to the peritoneum. In one patient, the mixed histotype was retained in the synchronous PM, but the tubular component only was found in the metachronous PM. In 2 out of 3 primary tumors of mucinous histotype, only the tubular (1/2) or the PCC (1/2) component metastasized.

An almost perfect concordance between primary tumors and paired PM was observed for E-Cadherin and p53, with an overall discordance rate between primary tumor, synchronous and/or metachronous PM of 5.4% and 1.4% for E-cadherin expression and p53 status, respectively.

A conversion from HER2-overexpressed primary tumor to HER2 not overexpressed metastasis was observed in 1/64 synchronous and 1/14 metachronous PM, with an overall discordance rate of 2.7% (2/74 patients). When scoring HER2 as zero/low/high, the overall discordance rate raised to 27.0% (20/74 patients), with 17/64 discordant synchronous PM and 4/14 discordant metachronous PM. All metachronous PM were HER2-zero. No statistically significant association was found between the homogeneous versus heterogeneous HER2 staining pattern and negative conversion of PM. Among the two primary HER2-overexpressed tumors that underwent negative conversion in PM, one exhibited a homogeneous staining pattern, while the other displayed a heterogeneous staining pattern.

**Fig. 2:**
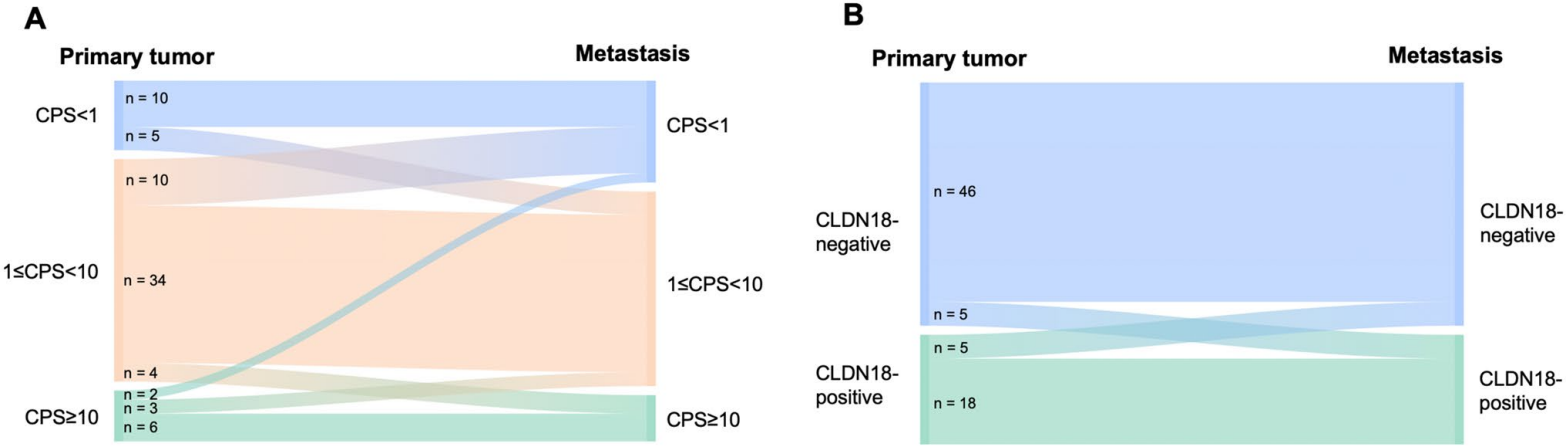
**A** PD-L1 and **B** CLDN18 expression in primary tumors (*n* = 74) and paired peritoneal metastases (PM). Cases with both synchronous and metachronous PM were considered only once; when the expression differed between synchronous and metachronous PM, the discordant case was considered. *PD-L1* programmed cell death ligand 1, *CPS* Combined Positive Score, *CLDN18* Claudin18

Regarding PD-L1 expression, the prevalence of negative cases (CPS < 1) increased from 20.3% in primary tumors to 29.7% and 28.6% in distant synchronous and metachronous PM, respectively. The overall discordance rate found considering CPS < 1, 1 ≤ CPS < 10, and CPS ≥ 10 as thresholds was 32.4% (24/74 patients), with negative conversion occurring more frequently than positive conversion. The discordance rate for synchronous and metachronous PM was 28.1% and 50.0%, respectively, but no significant difference was found in the number of discordant cases (*p*-value = 0.13). When considering CPS ≥ *vs* < 1, the discordance between primary G/EGJ and PM was 23.0%. All PM were concordant with the primary tumor for both MMR and EBER status. Changes in PD-L1 expression between primary tumors and paired PM are shown in Fig. 2A.

### Comparison of CLDN18 expression between primary gastric and esophago-gastric junction carcinomas and paired PM, and correlation with HER2 and PD‑L1 expression

CLDN18 was positive in 23/74 (31.1%) primary tumors and in 24/78 PM (30.8%; 32.8% in synchronous PM and 21.4% in metachronous PM). No statistically significant difference concerning CLDN18 positivity was found between primary tumors and PM and between synchronous and metachronous PM. In addition, CLDN18 was positive in 31.4% of patients with HER2-not overexpressed primary tumors and in 28.6% of patients with HER2-not overexpressed and PD-L1 CPS < 1 (*i.e.*, double-negative) primary tumors.

The discordance rate for synchronous and metachronous PM was 12.5% and 14.3%, respectively. The overall discordance rate between primary tumor, synchronous and/or metachronous PM was 13.5%, with 5/74 cases each of positive and negative conversion. Changes in CLDN18 expression between primary tumors and paired PM are shown in Fig. 2B. As for HER2, no statistically significant association was found between the homogeneous versus heterogeneous CLDN18 staining pattern and negative conversion of PM. Among the five primary CLDN18-overexpressed tumors that underwent negative conversion in PM, one exhibited a heterogenous staining pattern, while the other four displayed a heterogeneous staining pattern.

Table 2 summarizes the differences in morphology, molecular features and expression of predictive biomarkers between primary tumors and paired synchronous and metachronous PM.

**Table 2:** Comparison of morphology, molecular features and expression of predictive biomarkers between 74 primary gastric and esophago-gastric junction carcinomas and paired synchronous and metachronous peritoneal metastases

	All Metastases (*n* = 74)^‡^		Synchronous Metastases (*n* = 64)*		Metachronous Metastases (*n* = 14)*		*p*-value (Fisher’s exact test)^£^
	Discordance rate (95% CI)	Cohen’s κ	Discordance rate (95% CI)	Cohen’s κ	Discordance rate (95% CI)	Cohen’s κ	
Histotype	23.0% (13.4–32.6)	0.63	21.9% (11.7–32.0)	0.65	28.6% (4.9–52.2)	0.53	0.73
p53 (aberrant/wild-type)	1.4% (0–4.0)	0.97	1.6% (0–4.6)	0.97	0% (0–0)	1.00	1
E-Cadherin (lost/retained)	5.4% (0.3–10.6)	0.84	3.1% (0–7.4)	0.90	14.3% (0–32.6)	0.66	0.15
MMR (MMRd/MMRp)	0%	1.00	0%	1.00	0%	NA^†^	1
HER2 (overexpressed/not overexpressed)	2.7% (0–6.4)	0.65	1.6% (0–4.6)	0.79	7.1% (0–20.6)	0.00	0.33
HER2 (zero/low/high)	27.0% (16.9–37.1)	0.20	26.6% (15.7–37.4)	0.24	28.6% (4.9–52.2)	0.00	1
EBER (positive/negative)	0%	1.00	0%	1.00	0%	NA^†^	1
PD-L1 (CPS < 1/1 ≤ CPS < 10/ CPS ≥ 10)	32.4% (21.8–43.1)	0.41	28.1% (17.1–39.1)	0.50	50.0% (23.8–76.2)	0.15	0.13
CLDN18(positive/negative)	13.5% (5.7–21.3)	0.69	12.5% (4.4–20.6)	0.72	14.3% (0–32.6)	0.58	1

*MMR* Mismatch Repair, *MMRp* MMR proficient, *MMRp* MMR deficient, *CPS* Combined Positive Score, *CLDN18* Claudin 18, *EBER* Epstein–Barr virus–encoded small RNA, *PD-L1* programmed cell death ligand 1

^‡^When the biomarker expression differed between synchronous and metachronous metastases, the discordant case was considered

^*^For four patients one synchronous and one metachronous metastasis were available

^†^Cohen’s κ undefined because a single class is represented

^£^Calculated as difference in discordant rates between synchronous and metachronous metastases

### Comparison of morphology, molecular features and expression of predictive biomarkers in patients with both synchronous and metachronous PM

Regarding patients with both synchronous and metachronous PM, a complete concordance between primary tumors and both synchronous and metachronous PM was found for MMR, E-cadherin, CLDN18, p53, EBER and HER2 scored as overexpressed/ not overexpressed.

The histotype was concordant between the primary tumor and both PM in 2 cases, while in one case it was mixed in the primary tumor and PC-NOS in both PM and in another one it was discordant only in the metachronous PM (conversion from mixed to tubular histotype).

In two patients HER2 scored as zero/low/high was concordant between the primary tumor and the metachronous PM (HER2-zero) and discordant in the synchronous PM (HER2-low), while in one case the primary tumor was HER2-zero and both metastases were HER2-low.

In one patient the CPS was ≥ 1 and < 10 in the primary tumor and ≥ 10 in both PM.

### Implications of neoadjuvant therapy

Of the patients included in the study, 14/74 (18.9%) received neoadjuvant therapy. Among these, one case was pT1 for tumor extent, while all the other cases were pT3 or higher. None of the patients who received neoadjuvant therapy showed discordance for CLDN18 and HER2 expression, while 5/14 were discordant for PD-L1 expression. In 4/14 cases there was a conversion from 1 ≤ CPS < 10 to CPS < 1, and in 1/14 there was a conversion from 1 ≤ CPS < 10 to CPS ≥ 10. However, no significant difference was found in discordance rates between patients who received neoadjuvant therapy and those who did not.

### Combinations of concordant and discordant biomarkers

**Fig. 3:**
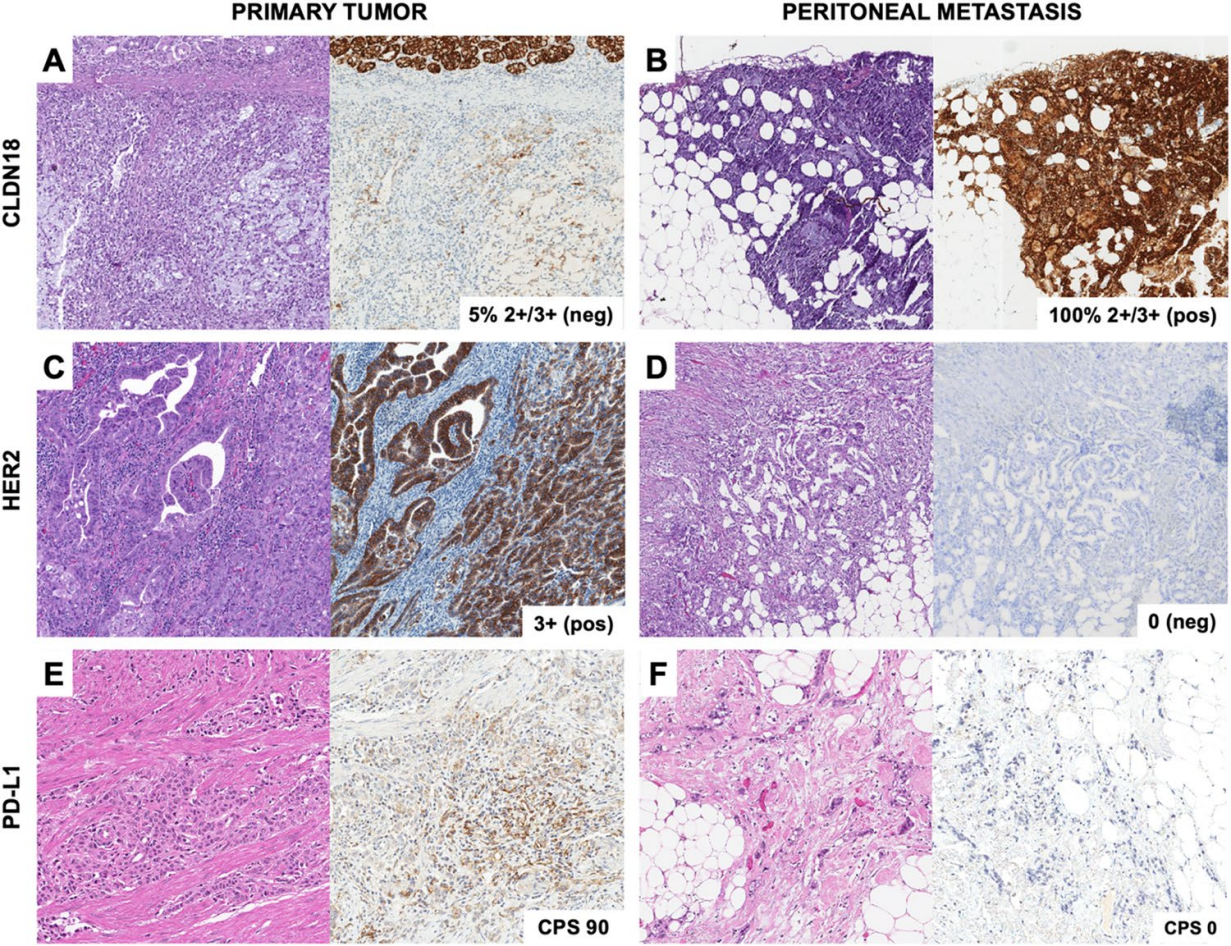
Representative images of discordant biomarkers’ status between primary tumor and matched peritoneal metastasis: **A** Claudin 18 (CLDN18)-negative primary tumor and **B** CLDN18-positive peritoneal metastasis, **C** HER2-overexpressed (3 +) primary tumor and **D** HER2-not overexpressed (0) peritoneal metastasis, **E** PD-L1 Combined Positive Score (CPS) 90 in primary tumor and **F** PD-L1 CPS 0 in peritoneal metastasis

In most patients, (42/74, 56.8%) all predictive biomarkers (*i.e.*, HER2, EBER, PD-L1, CLDN18 and MMR) were concordant between primary tumors and paired PM. In 28/74 patients (37.8%) one predictive biomarker was discordant, while 4/74 patients (5.4%) had two discordant predictive biomarkers. Combinations of concordant and discordant biomarkers found in each patient are shown in Fig. 1B. Examples of discordant biomarkers between primary tumor and peritoneal metastasis are shown in Fig. 3**.**

## Discussion

This study seeks to examine the expression patterns of established and emerging predictive biomarkers in G/EGJ with PM and to assess the concordance of biomarker expression between primary tumors and PM. The main findings can be summarized as follows: (i) G/EGJ with PM have specific morpho-molecular features, (ii) PD-L1 has the lowest concordance between primary tumor and PM among established biomarkers in G/EGJ; (iii) CLDN18 shows substantial concordance between primary G/EGJ and PM and could be a promising biomarker in G/EGJ with PM.

It is well established that poorly cohesive gastric carcinomas have a higher tendency towards peritoneal dissemination [20–23]. In fact, the majority of primary G/EGJ of our cohort were of poorly cohesive histotype or were partially composed of poorly cohesive tumor cells. Overall, primary G/EGJ with PM had a low prevalence of MMRd (4.1%), EBER positivity (1.4%) and HER2 overexpression (5.4%). According to The Cancer Genome Atlas (TCGA) analysis [24], MSI and EBV-associated GCs account for 22% and 8% of GCs, respectively. Furthermore, a previous study by Angerilli et al*.* [11], which involved a similar cohort of G/EGJ in terms of ethnicity and institutions, found higher frequencies of MMRd (11.8%), EBER positivity (4.5%), and HER2 overexpression (11.9%). Conversely, the prevalence of PD-L1 positivity in primary G/EGJ of the present cohort was comparable to that of Angerilli et al*.* [11] (79.7% *vs* 81.3%).

As for intrapatient heterogeneity of predictive biomarkers between primary G/EGJ and paired PM, MMR and EBER status achieved perfect concordance. Despite being subject to a semiquantitative scoring system and having high rates of intratumoral heterogeneity [25], HER2 achieved a good level of concordance, when scored as overexpressed/not overexpressed (*i.e.,* positive/negative). Instead, of all established predictive biomarkers in G/EGJ current diagnostic work-up, PD-L1 suffered the highest discordance between primary tumor and PM. The evidence regarding the discordance of PD-L1 expression between primary G/EGJ and paired metastases is still limited. Previous studies have found higher rates of PD-L1 expression in lymph nodes and distant metastases compared to primary tumors [26–28], but, in contrast to these findings, in our cohort the prevalence of PD-L1 negative cases (CPS < 1) increased from 20.3% in the primary tumor to 29.7% and 28.6% in distant synchronous and metachronous PM, respectively. The overall discordance rate found considering CPS of 1 and 10 as thresholds was 32.4%, with negative conversion occurring more frequently than positive conversion. Similarly, Massa et al*.* [29] reported a 23.1% discordance rate between primary tumors and distant metastases considering a threshold CPS of 5, with negative conversion occurring more often than positive conversion. These findings indicate that assessing PD-L1 status exclusively on peritoneal specimens may be unreliable. Thus, it is important to include primary tumor samples, such as biopsies or surgical specimens, when available, in the evaluation of patients with PM.

Recently, HER2-low status has gained the spotlight in gastric cancer, with several clinical trials evaluating the efficacy of antibody–drug conjugates. The targetability of HER2-low in gastric cancer, however, has yet to be established and further studies are needed to understand the clinicopathological and molecular features of this subgroup and enhance HER2 assessment methods, aiming to minimize interobserver variability [11, 30]. This is the first study evaluating the concordance of HER2 status, including HER2-low, between primary G/EGJ and paired PM. Here, we reported a discordance rate of 27% and a poor concordance overall. A recent study [31] conducted on several solid tumors reported a 16% discordance in a cohort of 25 cases of G/EGJ and paired metastases. Although the re-testing of metastatic recurrence in breast cancer has been integrated into current practice [32], in the case of recurrent G/EGJ, patients less frequently undergo surgery or re-biopsy. These results show that re-testing of HER2 status in the metastatic sample, when feasible, may help therapeutic decision-making.

The present study demonstrated that CLDN18 positivity in G/EGJ with PM was 31.1% at 75% cut-off in primary tumors, in agreement with a recent study [33] on gastric cancer with PM which reported a positivity rate of 28.6%. A prior study [34] demonstrated that CLDN18 expression was significantly reduced in gastric cancer cases with PM compared to those without. This aligns with our findings, showing a lower CLDN18-positive ratio in this study compared to the FAST and SPOTLIGHT studies. Interestingly, comparable CLDN18 positivity rates were observed in patients with HER2-not overexpressed (31.4%) and with HER2-not overexpressed and PD-L1 CPS < 1 (28.6%) primary tumors, indicating that CLDN18 could serve as a potential therapeutic target in double-negative patients with PM. Because CLDN18.2 functions as a tight junction protein, the loss of CLDN18.2 might trigger epithelial-mesenchymal transition, facilitating the spread of cancer cells into the peritoneal cavity. A recent study [33] found a slightly reduced CLDN18 expression in PM, but the concordance rate of CLDN18 status between primary tumor and PM was more than 75%. In our cohort, we observed no difference regarding CLDN18 status between primary tumor and PM with a good concordance overall (86.5%).

This study has several limitations, including its retrospective design and the absence of data on survival outcomes and adjuvant therapy. Additionally, the cohort included a limited number of HER2-overexpressed, MMR-deficient, and EBER-positive tumors. The number of metachronous peritoneal metastases was also lower than that of synchronous metastases, as these are typically resected during routine clinical practice. Furthermore, the inclusion of four cases with biopsy specimens from the primary tumor only may represent a limitation, as these samples may not fully capture tumor heterogeneity compared to full-slide specimens. However, this reflects a real-world situation where biopsies are often the only available material for clinical decision-making.

In conclusion, to the best of our knowledge, this is the first study to compare the expression of established and emerging biomarkers between primary tumors and PM. Our data suggest that G/EGJ with PM exhibit distinct morphologic and molecular characteristics, along with clinically significant molecular spatial heterogeneity associated with peritoneal dissemination. Among the established biomarkers, MMR, EBER, and HER2 demonstrated strong concordance between primary G/EGJ and PM, whereas PD-L1 showed greater heterogeneity. Importantly, CLDN18.2 emerges as a promising therapeutic target for G/EGJ patients with PM. As novel biomarkers, such as HER2-low and CLDN18.2, are integrated into clinical practice, addressing heterogeneity between primary tumors and metastases is becoming increasingly critical.

## Supplementary Information

Below is the link to the electronic supplementary material.

Supplementary file1 (DOCX 18 KB)

## Data Availability

The data that support the findings of this study are available on request from the corresponding author.
