# Supplementary material for: Heterogeneity of predictive biomarker expression in gastric and esophago-gastric junction carcinoma with peritoneal dissemination
Source: Gastric Cancer. 2025 Apr 9;28(4):569–78. doi: 10.1007/s10120-025-01609-7 (PMC12174275; doi:10.1007/s10120-025-01609-7)
Supplement: Supplementary file 1 — Supplementary file1 (DOCX 18 KB) [file 10120_2025_1609_MOESM1_ESM.docx]

**Supplementary Table 1.** Clinico-pathologic features of 74 patients with gastric and esophago-gastric junction carcinomas with peritoneal dissemination.

| Sex | Male | 40/74 (54.1%) |
| --- | --- | --- |
|  | Female | 34/74 (45.9%) |
| Age (median [range]) years | | 66.5 (29-89) |
| Site | Gastroesophageal Junction | 9/74 (12.2%) |
|  | Corpus/fundus | 12/74 (16.2%) |
|  | Antrum/angulus | 43/74 (58.1%) |
|  | Linitis Plastica | 7/74 (9.5%) |
|  | Anastomosis | 3/74 (4.1%) |
| Histotype | Poorly Cohesive | 34/74 (45.9%) |
|  | - PC-SRC  - PC-NOS/SRC  - PC-NOS | 9/74  6/74  19/74 |
|  | Mixed | 28/74 (37.8%) |
|  | Tubular | 8/74 (10.8%) |
|  | - Low grade  - High grade | 4/74  4/74 |
|  | Mucinous | 3/74 (4.1%) |
|  | Adenosquamous | 1/74 (1.4%) |
| pT^a^ | pT1 | 1/70 (1.4%) |
|  | pT2 | 0/70 (0%) |
|  | pT3 | 16/70 (22.9%) |
|  | pT4 | 53/70 (75.7%) |
|  | - pT4a  - pT4b | 39/70  14/70 |
| pN^b^ | pN0 | 8/69 (11.6%) |
|  | pN1 | 9/69 (13.0%) |
|  | pN2 | 16/69 (23.2%) |
|  | pN3 | 36/69 (52.2%) |
|  | - pN3a  - pN3b | 16/69  20/69 |
| Neoadjuvant therapy | Yes | 14/74 (18.9%) |
|  | No | 60/74 (81.1%) |

^a^Information not available for 4 cases

^b^Information not available for 5 cases

Abbreviations: PC= Poorly cohesive; SRC: Signet-Ring cell, NOS: Not otherwise specified.
